# ASSOCIATIONS BETWEEN HEARING LOSS AND FUNCTIONAL BRAIN CONNECTIVITY AMONG DEMENTIA-FREE OLDER ADULTS

**DOI:** 10.1093/geroni/igae098.1072

**Published:** 2024-12-31

**Authors:** Kening Jiang, Nicholas Reed, Anja Soldan, Frank Lin, Marilyn Albert, Jennifer Deal, Corinne Pettigrew

**Affiliations:** Johns Hopkins University, Baltimore, Maryland, United States; Johns Hopkins University, Baltimore, Maryland, United States; Johns Hopkins University, Baltimore, Maryland, United States; Johns Hopkins University, Baltimore, Maryland, United States; Johns Hopkins University School of Medicine, Baltimore, Maryland, United States; Johns Hopkins University, Baltimore, Maryland, United States; Johns Hopkins University School of Medicine, Baltimore, Maryland, United States

## Abstract

Hearing loss is associated with cognitive decline and mood disorders, potentially through functional changes in the brain, as auditory deprivation affects higher-order sensory, attentional, and emotional processing. This study examined the cross-sectional association between hearing loss and functional connectivity, in 141 dementia-free participants from the BIOCARD Study (Median age=71 years). Resting state functional MRI data were processed to construct seven large-scale functional networks: frontoparietal control, default mode, dorsal attention, limbic, salience/ventral attention, somatomotor, and visual. Hearing loss was assessed by pure-tone audiometry. Better-ear four-frequency pure-tone average (PTA: average of hearing thresholds at 0.5, 1, 2, and 4 kHz) in decibels hearing level (dB HL) was analyzed categorically (hearing loss: >25 dB HL vs. normal hearing: ≤25 dB HL) and continuously after scaling to per 10 dB HL higher (worse). We used linear regression models, adjusting for age, sex, education, time between functional connectivity and hearing assessments (-3 to 4.5 years), APOE ε4, vascular risk factors, and diagnostic status. Hearing loss was associated with lower connectivity in the salience/ventral attention network (-0.02, 95% confidence interval [CI]: -0.05, -0.00). After restricting the analyses to 102 cognitively unimpaired (CU) participants, the association was stronger (-0.04, 95% CI: -0.07, -0.01). We also found lower connectivity in the limbic network (-0.04, 95% CI: -0.07, -0.00) associated with per 10 dB HL worse hearing among CU participants. Our study adds to the limited evidence in hearing loss-functional connectivity association. Disrupted functional connectivity in salience/ventral attention and limbic structures might underlie cognitive impairment related to hearing loss.

